# Exposures to Dental Products, Stomatological Preparations, and in Dental Care and Toothache reported to the PIC Erfurt (1997 to 2017)

**DOI:** 10.1038/s41598-020-65079-w

**Published:** 2020-05-15

**Authors:** Beate Budenz, Michael Deters, Dagmar Prasa, Helmut Hentschel

**Affiliations:** Poisons Information Centre (PIC), Erfurt, Germany

**Keywords:** Drug discovery, Oral analgesics

## Abstract

We examined human exposures to dental products (EDP), stomatological preparations (ESP), and in the context of dental care (EDC) or toothache (ETA) registered by the Poisons Information Centre (PIC) Erfurt from 1997 to 2017. Dental products like dental technical and filling materials belong to medical devices. Stomatological preparations were classified according to the ATC code and symptom severity to the Poisoning Severity Score (PSS). In total, 156 cases of EDP (136 cases with different tooth filling materials), 1167 cases of ESP (55.6% fluoride containing products), 979 cases of EDC, and 331 cases of ETA were registered. Symptom severity in EDP and ESP were asymptomatic or mild. In ETA and EDC, however, 35 cases with moderate and 5 cases with severe symptoms were detected. 5 moderate and 3 severe cases were caused by prolonged paracetamol overdose. Severe bleeding occurred following tooth extraction in a 41 year-old phenprocoumon treated patient after self-medication with acetylsalicylic acid and metamizole. Gingival injection of lidocaine plus epinephrine in a 37 year-old healthy woman resulted in severe bradycardia and cardiac arrest. Acute toxicity of EDP and ESP appears to be low. Prolonged paracetamol overdose because of toothache, and some dental treatment can result in severe symptoms.

## Introduction

Dental products belong to the medical devices and are classified according to their hazard potential, location of use in the human body, duration and method of application in class I (e.g. instruments, impression material and tray, and dental technological material), IIa (fillings, denture relining, clamp materials), IIb (e.g. dental implants, X-ray apparatus), and III (absorbable bone material)^1^. Stomatological preparations belong to therapeutic drugs and are targeted on the local dental treatment of mouth and throat diseases. Furthermore, they are used for the treatment of aphthae of heterogenous origin and different forms of gingivitis and stomatitis, lichen ruber mucosae, oral candidiasis, bacterial and viral infections. In this context, a numerous amount of different substances like antimycotics, antibiotics, antiseptics, glucocorticoids, topical anaesthetics, and other substances for local treatment are applied^2^. Additionally, in the context of toothache and dental care a huge number of different substances are used that belong neither to dental products nor to stomatological preparations (e.g. conduction anaesthetics, analgesics, antibiotics, antiphlogistics)^1,3^. These substances are either used by the patients without any control by the dentist or they are applied or prescribed by a dentist during or after dental treatment. We sought to examine cases of adverse human exposure to dental products (EDP), stomatological preparations (ESP), and in the context of dental care (EDC) and toothache (ETA) as information on this subject is lacking.

## Materials and Methods

### Data source

The Poisons Information Centre (PIC) Erfurt serves a population of 10.1 million inhabitants in four federal states (Mecklenburg–Western Pomerania, Saxony, Saxony–Anhalt, and Thuringia) in Germany. All calls regarding EDP, ESP, EDC, and ETA registered by the PIC Erfurt from 1997 to 2017 were analysed retrospectively. Because the study was retrospective and only anonymized data of patients were used, according to the §15 of the Medical Association’s professional code of conduct in Thuringia no vote of the Ethic Commission of the State Chamber of physicians was necessary (written confirmation on November 18, 2019). The conduct of the study followed the guidelines according to the Declaration of Helsinki. Only 1% of all cases were followed up to a known outcome.

### Classification of substances, age groups, and symptom severity

Fillings, dental prosthesis, brace, and implants were grouped in the category of dental products. Stomatological preparations were classified according to the “Anatomical Therapeutic Chemical (ATC) classification system“ of the WHO^4^. EDC and ETA cases were detected by text filters in our data base. For further selection of ETA cases a filter for analgetics according to their ATC-Code was used. Finally, all these cases were checked for plausibility. Data were evaluated regarding substances involved, patient age groups, and symptom severity. Age groups were: baby (<1 year), toddler (1 to 5 years), schoolchild (6 to 13 years), child of unknown age (younger than 14 years), adolescent (14 to 17 years), middle aged adult (18 to 64 years), elderly (older than 64 years), adult of unknown age (older than 17 years), age unknown. The symptom severity was classified from 0 to 3 according to the Poisoning Severity Score (PSS)^5^.

### Statistics

The 95% confidence intervals (CI_95_) for differences of the relative frequencies were calculated by approximation to Gaussian distribution for big control samples^6^.

## Results

**Figure 1:**
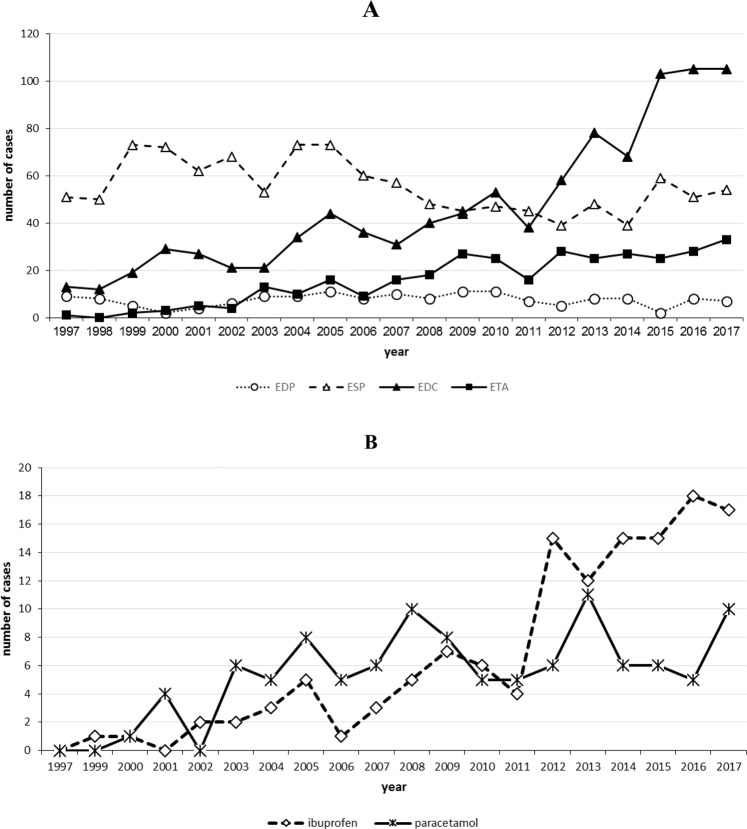
**(A)** Number of cases of human exposures to dental products (EDP), stomatological preparations (ESP), and in the context of dental care (EDC) and toothache (ETA) **(B)** particularly to paracetamol and ibuprofen reported to the PIC Erfurt from 1997 to 2017.

**Figure 2:**
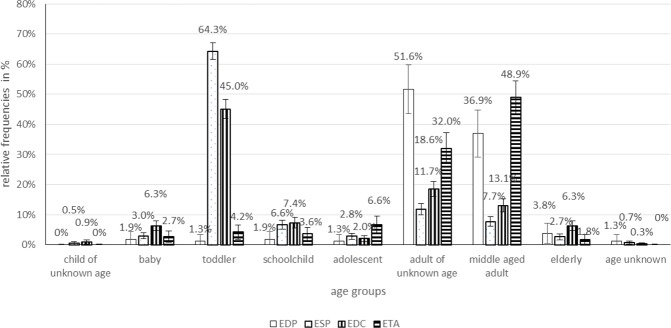
Relative frequencies in % +/− 95% confidence intervals (CI_95_) for differences of age groups in cases of human exposures to dental products (EDP), stomatological preparations (ESP), and in the context of dental care (EDC) and toothache (ETA) reported to the PIC Erfurt from 1997 to 2017.

**Figure 3:**
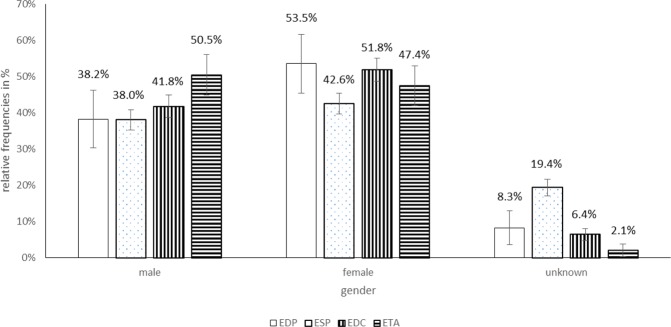
Relative frequencies in % +/− 95% confidence intervals (CI_95_) for differences of gender groups in cases of human exposures to dental products (EDP), stomatological preparations (ESP), and in the context of dental care (EDC) and toothache (ETA) reported to the PIC Erfurt from 1997 to 2017.

**Figure 4:**
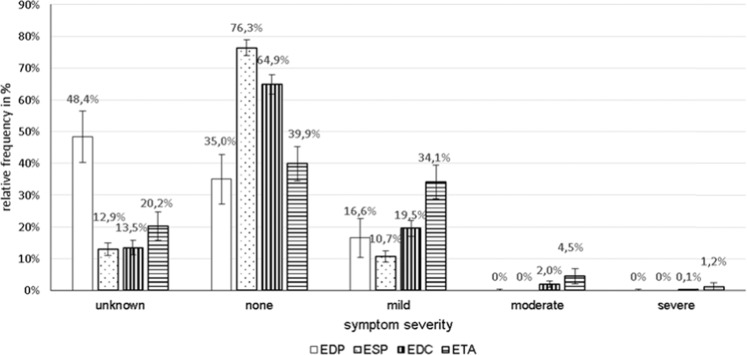
Relative frequencies in % +/− 95% confidence intervals (CI_95_) for differences of symptom severity in cases of human exposures to dental products (EDP), stomatological preparations (ESP), and in the context of dental care (EDC) and toothache (ETA) reported to the PIC Erfurt from 1997 to 2017.

In total, 156 EDP cases (136 cases of them with different tooth filling materials), 1167 ESP (649 cases of them with fluoride-containing products), 979 EDC, and 331 ETA cases were registered. While no clear tendency in the frequency of EDP and ESP cases could be observed, EDC and ETA cases increased from 13 and 1 in 1997 to 105 and 33 in 2017, respectively (Fig. 1). EDP cases mainly occurred in adults of unknown age (51.6%) and middle aged adults (36.9%). In ESP and EDC cases, however, the proportion of toddlers with 64.3% and 45.0% was the highest of all age groups (Fig. 2). ETA cases most often were seen in middle aged adults (48.9%) (Fig. 2). The proportion of female to male persons was elevated in EDP and EDC cases and balanced in ESP and ETA cases (Fig. 3). Symptom severity in EDP and ESP cases were asymptomatic or mild (Fig. 4). In EDC and ETA cases, however, 35 cases with moderate (6 cases with local anaesthetics (4 articaine, 2 unknown) plus epinephrine, 5 cases with prolonged paracetamol overdose) and 5 cases with severe symptoms (3 cases with prolonged paracetamol overdose) were detected (Fig. 4). In one case, severe bleeding occurred after extraction of two teeth in a man under phenprocoumon therapy, who treated himself with unknown amounts of acetylsalicylic acid and metamizole over two days because of toothache. In another case, gingival injection of lidocaine plus epinephrine in a 37-year old healthy woman by a dentist resulted in severe bradycardia and cardiac arrest (Table 1).

**Table 1:** All cases of human exposures in the context of dental care (EDC) and toothache (ETA) with severe symptoms reported to the PIC Erfurt from 1997 to 2017.

Case	Drug	Circumstances of exposure	Patient	Symptoms	Treatment
1	Phenprocoumon, metamizole,and acetylsalicylic acid	Tooth extraction after intake of metamizole and acetylsalicylic acid over 2 days because of toothache by a man under phenprocoumon therapy because of tachyarrhythmia absoluta and coronary heart disease	41-year old man	Massive bleeding after ambulant tooth extraction with obstruction of the upper airways and total inhibition of plasmatic coagulation	Intubation and administration of coagulation-promoting plasma and prothrombin complex concentrate. One day after tooth extraction surgical haemostasis, after six days installation of a tracheostoma
2	Lidocaine plus epinephrine	Iatrogenic gingival injection by a dentist	32-year old healthy woman	Collapse, severe bradycardia, probably cardiac arrest for a short time	Fast recovery after administration of Akrinor^®^ (cafedrine plus theodrenaline)
3	Ingestion of 4 g paracetamol corresponding to 73 mg/kg body weight/day over 3 days	Intake because of toothache	40-year old woman	Liver failure	Intravenous administration of acetylcysteine plus silibinin although already 3 days had been passed since paracetamol was ingested. The outcome is unknown
4	Ingestion of 12 g paracetamol corresponding to 146 mg/kg body weight within 12 h	Intake because of toothache	24-year old man	Liver failure	Intravenous administration of acetylcysteine plus silibinin, prearrangements for liver transplantation 2 days after paracetamol ingestion. After further 2 days improvement of liver function avoiding liver transplantation
5	Ingestion of 7.5 g paracetamol corresponding to 107 mg/kg body weight/day over 2 days	Intake because of toothache	Man of unknown age	Petechial bleeding, liver failure	Intravenous administration of acetylcysteine until liver enzymes were in the normal range. The outcome is unknown

## Discussion

### EDP

In the EDP cases of our study most often fillings out of amalgam, gold, palladium, plastic, materials for dental crowns and prosthetics like ceramic, caustics like calcium hydroxide, bonding and special adhesives were involved. In these cases either no or only mild symptoms were observed. Fillings are less under discussion because of their acute but of their chronic toxicity. In 2005, the Federal Institute for Drugs and Medical Devices (BfArM) stated that up to the current level of knowledge at that time there was no evidence for any harm on health of patients with intact amalgam fillings^7^. According to the Regulation (EU) 2017/852 on mercury it is not allowed in the European Union to install amalgam fillings in children younger than 15 years as of 1^st^ July 2018. In pregnant and breast-feeding women they should only be used in medically justified exceptional cases^8^. Alloys out of gold and palladium are only corrodible to a minimal extent^9^. The use of dental alloys results in 0.2% of all cases in unspecific symptoms. The average frequency of allergic reactions to dental materials is described to be about 2.5‰^9^. During application of calcium hydroxide in concentrations up to 33% there is a risk of local chemical burns both for the patient and the dentist^9^. Official reports on the frequency of chemical burns within the scope of dental treatment do not exist. Chemical burns by hydrofluoric acid are especially dangerous because hydrofluoric acid is lipophilic and able to penetrate into the surrounding tissue very easily. A fluoride-induced decrease of the Ca^2+^ concentration in serum can result in tetany and cardiac arrhythmia^10^.

### ESP

In ESP cases mainly caries prophylactic agents like sodium fluoride plus vitamin D, antiinfectives and antiseptics for local oral treatment like acriflavine, hydrogen peroxide, and chlorhexidine as well as other agents for local oral treatment like teething oil and Dentinox^®^ (extract of chamomile flower plus lidocaine) were involved. The acute lethal dose of fluoride in adults lays between 32–64 mg/kg body weight. In children, a probably toxic dose of 5 mg/kg body weight can be assumed. Symptoms of fluoride poisoning are nausea, vomiting, abdominal pain, hypersalivation, lacrimation, headache, cold and clammy hands, general weakness with spasm and tetany^11^. The daily intake of 1.5 mg fluoride until the age of eight years can result in dental fluorosis, that might be cumbersome only for cosmetic reasons. A daily fluoride intake of 0.05 mg/kg body weight is not a health risk^11^. Acriflavine was used for the local treatment of stomatitis in children and adults as well as in dermal mycosis and wound therapy. The International Agency for Research on Cancer (IARC) classifies acriflavine as possible carcinogen^12^. For this reason, acriflavine-containing drugs are currently not used in Germany in human medicine. Hydrogen peroxide up to a concentration of 5% causes only dermal and mucosal irritation. After ingestion of hydrogen peroxide in a concentration between 10 and 30% nausea, haematemesis, violent pain, foam formation due to gas release with the risk of aspiration risk, meteorism, vertigo and tussive irritation were described^13^. The oral resorption of chlorhexidine is poor. Its use in concentrations up to 2% bears no health risk^13^. Teething oils are mostly essential oils out of dianthus buds, chamomile, lavender or oenothera. They can like other oils induce a chemical pneumonitis after aspiration. After oral application by parents or elderly siblings to babies laryngospasm with hypoxia, acidosis and generalized seizures were observed^13^. The toxicologic relevant ingredient of Dentinox-Gel N^®^ is lidocaine. After exceeding the single maximum dose in children of 4.5 mg/kg body weight, lidocaine was described to cause somnolence, agitation, bradycardia, and respiratory distress^14^. The Food and Drug Administration (FDA) is warning, that overdosing of lidocaine-containing teething products in a child can result in death^15^.

### EDC

In EDC cases, the ingestion of toothpaste was the most frequent scenario. In these cases no moderate or severe symptoms were observed. From the toxicologic point of view fluorides are the most important components in toothpaste. The ingestion of one tube tooth paste for adults (100 g) with fluoride concentration of 1000 ppm is not a health risk for an adult. If the same tube would be ingested by a 3-year old child with a body weight of 15 kg, however, the toxic dose of 5 mg/kg body weight would be exceeded by 33%. The ingestion of one tube tooth paste for children (50 g) and a fluoride concentration of 500 ppm by the same child (fluoride dosis 1.7 mg/kg body weight) would bear no health risk. Moderate and severe symptoms were only observed in EDC and ETA cases. 6 of 35 moderate cases and one sever case were seen after gingival administration of local anaesthetics. Systemic toxicity of local anaesthetics is well described in literature. Seizures were the most common presentation. The spectrum of presenting neurologic and cardiovascular symptoms and signs are broad. It is recommended, that all physicians who administer local anesthetics should be educated regarding the nature of systemic toxicity and contemporary management algorithms that include lipid emulsion therapy^16^.

### ETA

In ETA cases with moderate or severe symptoms, the prolonged overdose of paracetamol because of toothache was dominant. In children older than 6 years, the intake of paracetamol in a dose of 80 mg/kg bodyweight over 24 hours can result in irreversible liver and kidney injury^13^. In a previous study, a high correlation between ibuprofen- and paracetamol monoexposures and their respective package sales was shown^17^. The prescriptions of ibuprofen in Germany rose from 319 million defined daily doses in 2008 to 564 million in 2016 and decreased slightly 546 million in 2017. In our study, a similar time course of the yearly frequency of ibuprofen in ETA cases in the corresponding observation time was seen (Fig. 1B). In prescriptions by dentists, ibuprofen with a proportion of almost 78% to all nonsteroidal anti-inflammatory drugs is dominant and preferred because of less side effects^18^. The prescriptions of paracetamol continously decreased from 20 million defined daily doses in 2008 to 10 million in 2015 and remained at that level until 2017. Paracetamol has not been described by dentists in a mentionable amount since 2015^19^. In our study, the time course of the number of ETA cases with paracetamol showed no clear tendency (Fig. 1B) and seems not to be influenced by the changes in the presciptions of paracetamol by dentists. One possible explanation could be that paracetamol as an OTC drug needs no prescription and this worsens the medically controlled paracetamol intake by the patient. Therefore it seems so much more important that pharmacists inform their clients on the risks of paracetamol intake.

## Limitations

The study was retrospective. The data of the EDP, ESP, EDC, and ETA cases came from spontaneous enquiries (56.4% private persons and 38.0% medical experts) and are not representative for the average population. Because in many cases the practitioner or the hospital will only be contacted in case of uncomfortable symptoms, an underestimation of case numbers can be expected. The reliability of information about ingested doses on oral reports of medical staff, involved persons or their relatives was limited and analysis of involved substances was not performed in the most cases. The high proportion of exposures with unknown symptoms reduces the informative value of differences of symptom severity between the respective exposure groups. The low proportion of cases followed up to a known outcome may lead to underestimation of symptom severity.

## **Conclusions**

The acute toxicity of dental and stomatological products seems to be low. An elevated risk in EDC and ETA cases can be expected in case of prolonged paracetamol overdose because of toothache or in case of neglection of contraindications of therapeutic drugs (i.e. phenprocoumon) for the performance of some dentical therapeutic measures (e.g. tooth extraction, gingival administration of local anaesthetics). Although dentists do not prescribe paracetamol in a noteworthy amount since 2015 no reduction of ETA with paracetamol could be observed by the PIC Erfurt after that time. Because paracetamol is an OTC drug pharmacists play an important role in informing clients on the risks of paracetamol intake.
